# 

**Published:** 1872-06

**Authors:** 


### ARTICLE III.

### *Alabama Dental Association.*

The next meeting of the Alabama Dental Association will be held in Montgomery, on the third Wednesday in August, 1872, at which time it is hoped a full attendance of the profession of the State will be present, and also members of the profession generally are invited. It is the intention of the members of the Society to make it the most interesting meeting yet held. A number of essayists are appointed and any who may favor us with matter will be appreciated by the members of the Society. C. A. Walker, Rec. Sec'y.

### EDITORIAL DEPARTMENT.

*Meetings of Dental Associations.*—The Southern Dental Association meets at Richmond, Va., the last Tuesday in July, 1872, at 10 o'clock, A. M. President, Dr. F. Y. Clark; Vice-Presidents, Drs. J. B. Patrick, H. M. Grant, E. Floyd; Cor. Sec'ty., Dr. A. C. Ford; Rec. Sect'y., O. J. Bond.

American Dental Association meets at Niagara Falls, the first Tuesday in August, 1872. President, Dr. Geo. W. Cushing; Rec. Sect'y., Dr. M. S. Dean.

American Academy of Dental Science meets at Boston, in September, 1872. President, Dr. Daniel Harwood; Rec. Sect'y, Dr. L. D. Shepard; Cor. Sect'y, Dr. E. N. Harris. Address by Prof. P. H. Austen, M.D., D.D.S.

American Dental Convention meets at Boston, second Tuesday in August. President, Dr. Wm. Dutch; Rec. Sect'y., Dr. C. S. Hurlburt.

Virginia State Dental Society meets at Richmond, in November, 1872. President, Dr. H. M. Grant; Vice-Presidents, Drs. J. F. Thompson, W. B. Pleasants, A. B. Arthur; Sect'y., Dr. G. F. Keesee.

Eastern Ohio Dental Association meets at Salem, August 29, 1872. President, Dr. J. C. Whinery; Sect'y., Dr. D. B. McLain.

South Carolina State Dental Asssociation meets at Columbia, Tuesday, July 23d, 1872. Cor. Sec., *pro tem*, Thos. F. Moore.

